# Aromatase inhibitor treatment with an intravaginal device and its effect on pre-ovulatory ovarian follicles in a bovine model

**DOI:** 10.1186/1477-7827-11-97

**Published:** 2013-10-03

**Authors:** Jimena Yapura, Reuben J Mapletoft, Roger A Pierson, Jaswant Singh, Gregg P Adams

**Affiliations:** 1 Department of Veterinary Biomedical Sciences, Western College of Veterinary Medicine, University of Saskatchewan, Saskatoon, Saskatchewan, S7N 5B4, Canada; 2 Department of Large Animal Clinical Sciences, Western College of Veterinary Medicine, University of Saskatchewan, Saskatoon, Saskatchewan, S7N 5B4, Canada; 3 Department of Obstetrics, Gynecology and Reproductive Sciences, College of Medicine, University of Saskatchewan, Saskatoon, Saskatchewan, S7N 0W8, Canada

**Keywords:** Aromatase inhibitor, Letrozole, Ovarian function, Follicle development, Bovine

## Abstract

**Background:**

Letrozole, a non-steroidal aromatase inhibitor, prevents the body from producing its own estrogen. The objectives of the present study were to test the hypotheses that letrozole treatment, initiated prior to selection of the preovulatory dominant follicle, will induce the growth of more than one follicle to a pre-ovulatory size, and will delay ovulation.

**Methods:**

Post-pubertal beef heifers were given two luteolytic doses of PGF (12 h apart) and monitored by ultrasonography for ovulation. Five to eight days later, ovarian follicular wave emergence was synchronized by ultrasound-guided transvaginal follicular ablation (Day 0=wave emergence) and a luteolytic dose of PGF was given 60 and 72 h later. On Day 1, heifers were divided randomly into two groups (n=15/group) and an intravaginal device containing 1 g of letrozole or a blank device (control) was inserted. The intravaginal devices were removed on Day 7, or at the time of ovulation, whichever occurred first. Transrectal ultrasonography and blood sample collection were performed daily from the day of ablation to 12 days after subsequent ovulation.

**Results:**

The mean (+/-SEM) interval from device placement to ovulation was longer in letrozole-treated animals compared to controls (6.1+/-0.25 vs 5.1+/-0.26 days, respectively; P<0.01). Single dominant follicles were present in both groups. The day-to-day diameter profiles of the dominant follicles of the ovulatory wave were larger (P<0.05) and the maximum diameters greater in letrozole-treated heifers (14.6+/-0.51 vs 12.4+/-0.53 mm, respectively; P<0.01). The diameter profile of the corpus luteum (CL) that formed after treatment did not differ between groups; however, plasma progesterone concentrations were higher (P<0.01) in heifers treated with letrozole. Estradiol concentrations were reduced following letrozole treatment (P<0.05), although a preovulatory rise of estradiol occurred in both groups.

**Conclusions:**

Administration of letrozole with an intravaginal device during growth of the ovulatory follicle delayed ovulation by 24 h and resulted in the formation of a CL that secreted higher levels of progesterone. A sustained-release intravaginal device may be useful for the development of an aromatase inhibitor-based protocol to control ovulation for herd synchronization and to enhance fertility by increasing circulating progesterone concentrations during the first 7 days post-ovulation in cattle.

## Background

Estrogen-based protocols, as a treatment for synchronizing ovulation in cattle, have modernized breeding practices and allowed producers to reliably control the timing of ovulation, thus enabling efficient use of time, labour and resources by allowing pre-scheduled insemination. Estradiol-based protocols also allow wider application of superovulation and embryo transfer by enabling effective synchronization of follicular wave emergence [1-5]. However, increasing consumer sensitivity to the possible deleterious effects of estrogens in food and in the environment [6] has led to new regulations about the use of estrogenic products in livestock. The European Union has already banned the use of estrogenic products in food producing animals [7-10]. In United States [11] and Canada [12], estrogens cannot be used for synchronization of estrus except by prescription and custom-compounding. In 2007, New Zealand and Australia banned use of estrogens in lactating dairy animals [10]. These policies have created a void in methods to control reproductive function for breeding management in cattle.

Non-steroidal aromatase inhibitors prevent the body from producing its own estrogen. Letrozole, a non-steroidal aromatase inhibitor, is used as an adjuvant treatment for hormone-responsive breast cancer in post-menopausal women [13] and has been used as a fertility treatment for women undergoing assisted reproduction [14]. The putative effect of letrozole on ovarian function in women is through elevated follicle-stimulating hormone (FSH) secretion by removal of the negative feedback of estradiol [14]. Tests of this hypothesis in a bovine model, however, were not supportive of an effect on FSH. In cattle, a single intravenous dose given on Day 3 post-ovulation, or a 3-day regimen given on Days 1–3, 3–5 or 5–7 post-ovulation did not induce an elevation in circulating FSH concentration but did increase mean plasma luteinizing hormone (LH) concentrations. The effect on LH secretion resulted in a prolonged period of dominance of the extant dominant follicle and delayed emergence of the next follicular wave [15,16]. Further, a luteotrophic effect was inferred from the observation that heifers treated with letrozole for 3 days had larger corpora lutea following treatment. Similar results were observed when letrozole was prepared in an oil-based vehicle and administered intramuscularly [17].

To date, studies on the effects of letrozole on ovarian function in cattle have been focused on non-ovulatory follicular waves [15,16]. The present study was designed to determine the effect of an extended period of treatment with letrozole on the pre-ovulatory follicle in cattle. We hypothesized that letrozole treatment initiated before selection of the dominant ovulatory follicle and extended over the follicle growing phase will induce the development of more than one follicle to a preovulatory size, and delay ovulation. Additionally, we hypothesized that the CL resulting from ovulations after letrozole treatment will be larger and secrete more progesterone than those from control heifers.

## Methods

### Cattle

Hereford-cross beef heifers (n=30), 15 to 20 months of age and weighing between 235 and 405 kg (average 336 kg), were chosen from a herd of 51 heifers maintained in outdoor pens at the University of Saskatchewan Goodale Research Farm (52° North and 106° West). Heifers were fed alfalfa/grass hay and concentrate to gain approximately 1.3 Kg per day and had water ad libitum during the experimental period from October to December. Heifers were initially examined by transrectal ultrasonography (MyLab5 VET, Canadian Veterinary Imaging, Georgetown, Ontario Canada) to detect the presence of a CL (i.e., confirm post-pubertal status; [18]. Animal procedures were performed in accordance with the Canadian Council on Animal Care and were approved by University of Saskatchewan Protocol Review Committee.

### Treatments and examinations

Heifers in which a CL was detected were given two luteolytic doses of PGF (12 h apart) and monitored by ultrasonography for ovulation. Five to eight days later, the two largest ovarian follicles were ablated by transvaginal ultrasound-guided follicular aspiration to synchronize follicular wave emergence which was expected to occur 1 to 1.5 days later [19,20]. Prostaglandin (500 μg cloprostenol, Estrumate, Schering-Plough Animal Health, Pointe-Claire, QC, Canada) was given intramuscularly at 60 and 72 h after follicular ablation to induce regression of the CL and shift from a non-ovulatory to an ovulatory follicular wave [21]. At the time of follicular wave emergence (Day 0; i.e., 1.5 days after follicle ablation), heifers were assigned randomly to two groups and given an intravaginal device containing 1 g of letrozole (letrozole group, n=15) or a placebo (letrozole-free) intravaginal device (control group, n=15). Devices were inserted on Day 1 and were kept in place until Day 7 or until ovulation was detected, which ever occurred first.

Intravaginal devices were prepared using a Cue-Mate spine (Bioniche Animal Health, Bellville, ON, Canada) assembled with two blank (progesterone-free) silicone pods that were coated with a gel-based vehicle containing letrozole or vehicle only (control). The vehicle contained the following (all ingredients % w/w): letrozole 10%, gelatin 20% (Gelatin type B, Fisher Scientific, Pittsburgh, PA, USA), polymer 65% (prepared by mixing distilled water 68%, Poloxamer 188 12% and Poloxamer 407 20%, both from Spectrum Chemical, New Brunswick, NJ, USA) and distilled water 5%.

### Ovarian ultrasonography

The observations from ultrasound examinations were recorded on a sketch sheet in which each ovary and its structures (CL and follicles ≥ 4 mm in diameter) were represented by size and location [22]. Ovulation was defined as the disappearance of any follicle ≥8 mm between two consecutive daily examinations and was confirmed by the subsequent development of a CL [18]. Follicular wave emergence was defined as occurring 1.5 days after follicular ablation [19]. The dominant follicle of a wave was defined as the largest antral follicle of that wave [23].

### Collection of blood samples

Blood samples were collected by coccygeal venipuncture into 10 mL heparinized vacuum tubes (Becton Dickinson Vacutainer Systems, Franklin Lakes, NJ, USA). Samples were collected daily from pre-treatment follicular wave emergence (Day 0) to 12 days after the subsequent ovulation. In a subset of letrozole-treated animals (n=4), frequent blood samples were collected using an in-dwelling jugular catheter, as previously described [24], at the time of catheter placement and 0, 10, 20, 30 min, 1, 1.5, 2, 3, 4, 6, 8, 12 and 24 h after treatment for measurement of plasma letrozole concentration. Blood samples were centrifuged at 1500 × g for 20 min and plasma was separated and stored in plastic tubes at -20°C.

### Hormone assays

Plasma LH concentrations were determined in duplicate using a double-antibody radioimmunoassay (NIDDK-bLH4) [25,26]. The minimum and maximum values along the standard curve were 0.06 and 8 ng/mL, respectively. All samples were analyzed in a single assay; the intra-assay coefficient of variation was 11.4% for low reference samples (mean, 0.9 ng/mL) and 12.2% for high reference samples (mean, 2.1 ng/mL).

Plasma FSH concentrations were determined in duplicate using a double-antibody radioimmunoassay using NIDDK-anti-oFSH-1 primary antibody and expressed as USDA bovine FSH-Il units [25,26]. The minimum and maximum values along the standard curve were 0.12 and 16 ng/mL, respectively. All samples were analyzed in a single assay; the intra-assay coefficients of variation were 7.9 and 6.5%, for low (mean, 2.4 ng/mL) and high reference samples (mean 4.9 ng/mL), respectively.

Plasma concentrations of estradiol were determined using a commercial radioimmunoassay kit (Double Antibody Estradiol; Diagnostic Products Corp., Los Angeles, CA, USA). The procedure was carried out at the Department of Animal Health and Biomedical Sciences, University of Wisconsin–Madison, as previously described [27,28], with the following modifications: Standards (0.78–100 pg/mL) were prepared in steroid-free (charcoal-treated) bovine plasma. The standards (250 μL in duplicate) and plasma samples (500 μL in duplicate) were extracted with 3 mL of diethyl ether, frozen in a dry-ice/methanol bath, decanted into assay tubes, and dried overnight under a fume hood. The dried samples and standards were re-suspended with 100 μL of assay buffer (0.1% gelatin in PBS). The intra- and inter-assay coefficients of variation were 10.5 and 10.6% for high reference samples (mean 11.1 pg/mL), and 14.8 and 12.3% for low reference samples (mean 2.6 pg/mL), respectively. The sensitivity of the assay was 0.1 pg/mL.

Plasma progesterone concentrations were determined in duplicate using a commercial solid-phase radioimmunoassay kit (Coat-A-Count; Diagnostic Products Corporation, Los Angeles, CA, USA). The range of the standard curve was 0.1 to 40.0 ng/mL. All samples were analyzed in a single assay; the intra-assay coefficients of variation were 9.7% and 5.8 % for low- (mean, 1.7 ng/mL) and high-reference samples (mean, 18.7 ng/mL), respectively.

### Letrozole concentrations

Plasma letrozole concentrations were quantified using liquid chromatography tandem mass spectrometry (LCMS/MS), as described [16]. Briefly, letrozole was extracted from 250 mL plasma with 250 mL of 0.1M ammonium acetate followed by the addition of 5 mL methyl t-butyl ether (MTBE) and vortexed for 15 s. The organic layer was removed and transferred to a fresh 15 mL plastic tube and dried by gentle nitrogen gas flow. The dried extract was reconstituted in 1 mL of 100% ethanol. Separation was accomplished by HPLC (Agilent 1200; Agilent, Santa Clara, CA, USA) fitted with an analytical column (50 × 2.1 mm, 3 mm particle size; Betasil C18; Thermo Scientific, Waltham, MA, USA) operated at 35°C. Mass spectra were collected using a tandem mass spectrometer (SCIEX 3000; Applied Bioscience, Foster City, CA, USA) fitted with an electrospray ionisation source, operated in the negative ionisation mode. Quantification was performed using Analyst 1.4.1 software provided by SCIEX (Applied Bioscience). The minimum and maximum values along the standard curve were 0.25 and 500 ng/mL, respectively. The limit of quantification used in this method was 0.25 ng/mL and the mean recovery was 70%.

The following pharmacokinetic parameters were determined: *C*_*max*_ (maximum observed plasma concentration of letrozole), *t*_*max*_ (time to reach *C*_*max*_), *t*_*1*/*2*_ (terminal elimination half-life), *AUC* (area under the plasma letrozole concentration-time curve from zero to infinity calculated as *AUC*_*tlast*_). The concentration of letrozole in plasma as a function of time (C–t) data for each heifer was analyzed by non-compartmental techniques using a computer modeling program (WinNonLin Standard Edition Version 2.1, Pharsight Corporation, Mountain View, CA, USA). Peak letrozole concentration in plasma (C_max_) and time to peak letrozole concentration (t_max_) were determined using observed values. The apparent terminal rate constant (λ) was determined by linear regression of the last 6–8 points on the terminal phase of the logarithmic plasma concentration vs time curve. The area under the C_–t_ curve until the final plasma sample (AUC_last_) was determined using the linear trapezoidal rule. The total area under the curve extrapolated to infinity (AUC_0-∞_) was calculated by adding the C_last obs_/λ + AUC_last_. The terminal half-life (T_1/2λ_) was calculated as ln_2 ⁄λ_. The mean residence time (MRT) was calculated as the area under the moment curve extrapolated to infinity (AUMC_0-∞_) ⁄AUC_0-∞_. Systemic clearance (Cl_S_) was determined using the dose divided by AUC_0-inf_. The apparent volume of distribution (V_λ_/*f*) was calculated by clearance divided by λ. Absolute bioavailability was calculated by comparing letrozole AUC_last_ obtained using intravaginal devices to the AUC_last_ obtained after a single iv injection of letrozole (unpublished data), corrected by dose (Bioavailability = (AUC_last intravag_/Dose_intravag_)/(AUC_last iv_/Dose_iv_) × 100).

### Statistical analyses

Statistical analyses were done using the Statistical Analysis System software package (SAS Learning Edition 9.1, 2006; SAS Institute Inc., Cary, NC, USA). Time-series data (hormone concentrations, follicle and CL diameter profiles) were analyzed by repeated measures using the PROC MIXED procedure. The main effects were treatment (letrozole and control), time, and their interactions (noted in figures as treatment, day and treat*day, respectively). Single-point measurements (dominant follicle diameter at device placement, maximum diameter of extant dominant follicle, intervals from ablation to wave emergence, and from device placement to ovulation) were analyzed by t-tests. Individual time point comparisons between treatment groups were performed using least significant difference test. Significance was defined as P ≤ 0.05.

## Results

**Figure 1:**
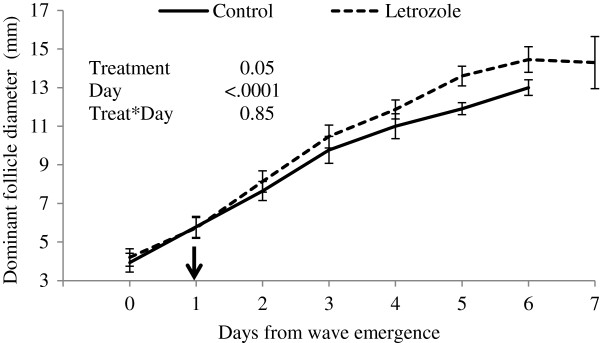
**Dominant follicle diameter profiles (mean±SEM).** Heifers were treated with a blank (control, n=15) or a letrozole-containing intravaginal device (letrozole, n=15). Devices were inserted on Day 1, indicated by the arrow (Day 0 = wave emergence).

The diameter of the dominant follicle at the time of intravaginal device placement on Day 1 (Day 0 = wave emergence) did not differ between groups (Table 1). The day-to-day diameter profile of the dominant follicle during treatment and the maximum diameter of the ovulatory follicle were larger in the letrozole-treated group (P=0.05 and P=0.01; respectively (Figure 1; Table 1; respectively). The interval from device placement to ovulation was longer in heifers treated with letrozole than in controls (P=0.01, Table 1). Single ovulation occurred in all heifers, regardless of treatment.

**Table 1:** **Effects of a letrozole**-**containing intravaginal device on ovarian function in heifers (mean**±**SEM)**

**End point**	**Control (n=15)**	**Letrozole (n=15)**	**P-value**
*Device placement to ovulation* (*days*)	5.1±0.26	6.1±0.25	<0.01
*Max*. *diameter of extant dominant follicle* (*mm*)	12.4±0.53	14.6±0.51	<0.01
*Dominant follicle diameter at device placement* (*mm*)	3.9±0.47	4.2±0.46	0.68

**Figure 2:**
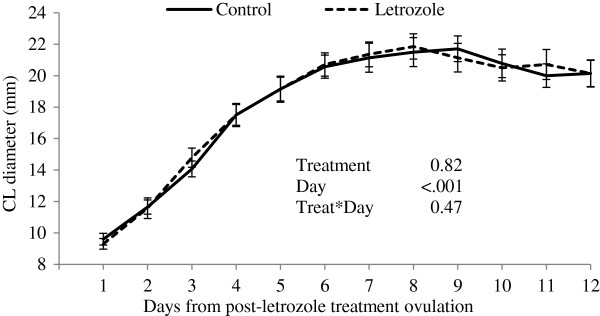
**Corpus luteum diameter profiles (mean±SEM) following post**-**treatment ovulation.** Heifers were treated with a blank (control, n=15) or a letrozole-containing intravaginal device (letrozole, n=15).

**Figure 3:**
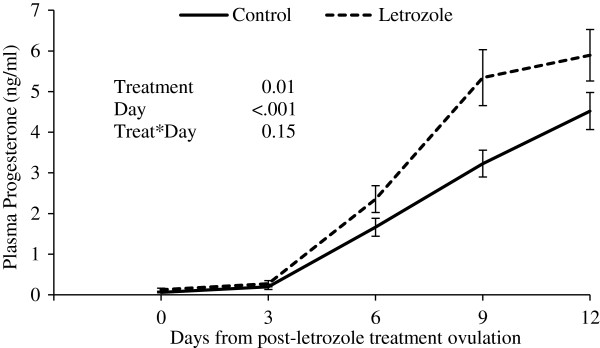
**Plasma progesterone concentrations (mean±SEM) following post**-**treatment ovulation.** Heifers were treated with a blank (control, n=15) or a letrozole-containing intravaginal device (letrozole, n=15).

Corpus luteum diameter profiles were not different between letrozole and control groups (P=0.82, Figure 2). However, progesterone concentrations were higher during the observational period (first 12 days post-ovulation) in the letrozole-treated heifers compared to control heifers (P=0.01, Figure 3).

**Figure 4:**
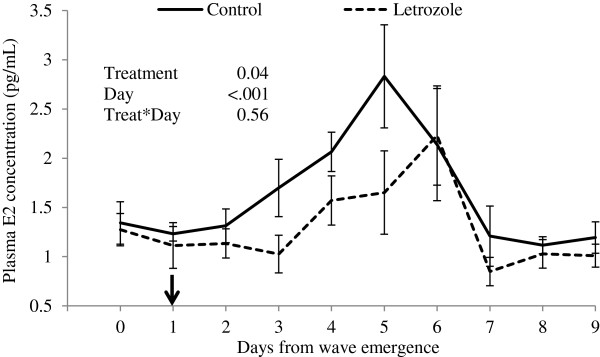
**Plasma estradiol concentrations (mean±SEM).** Heifers were treated with a blank (control, n=15) or a letrozole-containing intravaginal device (letrozole, n=15). Devices were given on Day 1 of the ovulatory wave (indicated by the arrow; Day 0 = wave emergence).

Plasma estradiol concentrations were lower in the letrozole-treated group than in the control group (P=0.04, Figure 4). Treatment with letrozole did not prevent a pre-ovulatory rise in estradiol but the rise was delayed in the letrozole group (Figure 4).

**Figure 5:**
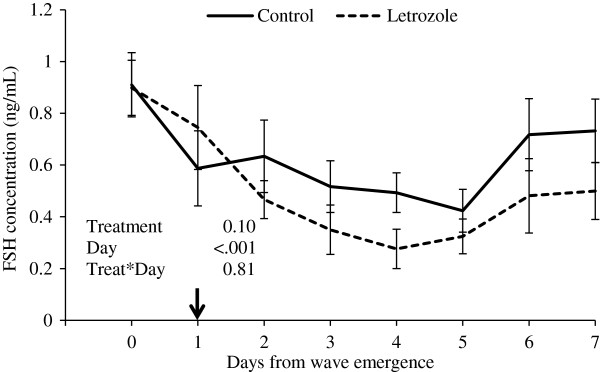
**Plasma FSH concentrations (mean±SEM).** Heifers were treated with a blank (control, n=15) or a letrozole-containing intravaginal device (letrozole, n=15). Devices were given on Day 1 of the ovulatory wave (Day 0 = wave emergence).

**Figure 6:**
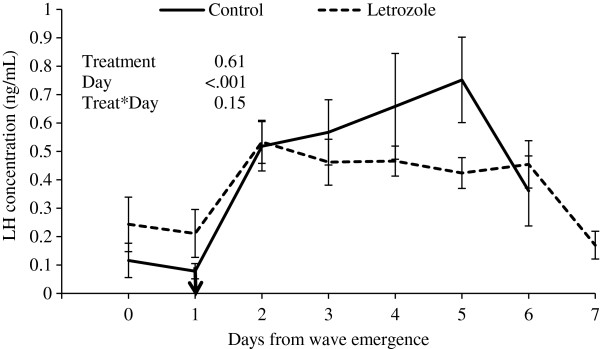
**Plasma LH concentrations (mean±SEM).** Heifers were treated with a blank (control, n=15) or a letrozole-containing intravaginal device (letrozole, n=15). Devices were given on Day 1 of the ovulatory wave (indicated by the arrow; Day 0 = wave emergence).

There was a tendency for lower plasma FSH concentrations in the letrozole group compared to the control group (P = 0.1; Figure 5). Mean plasma LH concentrations did not differ between groups (P = 0.61; Figure 6).

**Figure 7:**
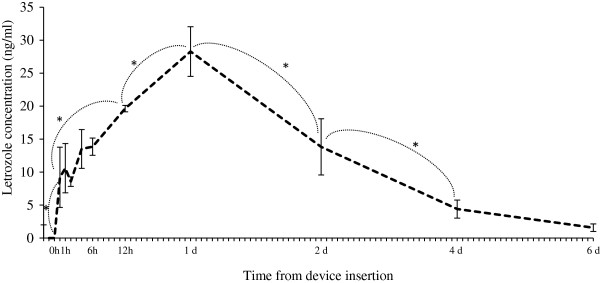
**Plasma letrozole concentration (mean±SEM).** Heifers (n=4) were given an intravaginal letrozole-releasing device for 6 days. *Between indicated time points, values differed (P≤0.05).

Plasma letrozole concentrations are shown in Figure 7. The half-life of letrozole in plasma was 33.3±4.56 h. Maximal concentrations in plasma (C_max_ 31.7±1.65 ng/mL) occurred at 24 h post-device insertion (Table 2). Additional letrozole pharmacokinetic parameters are summarized in Table 2.

**Table 2:** **Pharmacokinetics of a letrozole**-**containing intravaginal device in heifers**

**Parameter**	**Heifer 1**	**Heifer 2**	**Heifer 3**	**Heifer 4**	**Mean**	**SEM**
*Maximal concentration* (*C*_*max*_) (*ng*/*mL*)	32.4	35.3	27.3	31.7	31.7	1.65
*Half*-*life* (*T*_*1*/*2*_) (*hours*)	35.1	20.4	41.9	35.7	33.3	4.56
*Area under the curve* (*AUC*_*last*_) (*hours x ng*/*mL*)	3538.1	2697.7	2583.1	1698.0	2629.2	376.39
*Volume of distribution* (*V*_*z*_/*f*) (*L*/*kg*)	6.2	4.9	9.9	13.2	8.5	1.87
*Systemic clearance* (*Cl*_*S*_) (*L*/*hour*/*kg*)	0.1	0.2	0.2	0.3	0.2	0.03
*Mean residence time* (*MRT*) (*hours*)	71.5	56.2	65.5	48.7	60.5	5.03
*Bioavailability*					16%	

## Discussion

Previous studies of the effects of letrozole on ovarian function in cattle were focused on the non-ovulatory portion of the estrous cycle in cattle [15-17]; hence, the effect of letrozole treatment on pre-ovulatory follicles was the subject of the present study. Letrozole treatment during the pre-ovulatory follicular wave resulted in a greater diameter of the ovulatory follicle. This observation is consistent with the results of previous studies in which larger dominant follicles were observed when letrozole treatment was administered during non-ovulatory follicular waves [15-17]. The hypothesis that letrozole treatment initiated prior to the onset dominant follicle selection would result in multiple ovulatory follicle development [15] was not supported by the results of the present study; single ovulations were detected in both groups. However, ovulation was delayed by 24 h in the letrozole-treated group. The larger ovulatory follicle diameter observed in the letrozole-treated heifers may have been affected by the length of the growing phase of these follicles due to delayed ovulation. However, ovulatory dominant follicle diameters in the letrozole-treated group were already larger than the control group when compared 5 days after initiation of treatment (Day 6 post-wave emergence). The stimulus driving the accentuated follicular growth is unclear. However, daily sampling has limitations when attempting to interpret gonadotropin concentrations and its correlation to ovarian dynamics. Perhaps changes in gonadotropin pulse-frequency would have been detected using more frequent sampling.

Estradiol concentrations were reduced following treatment with letrozole-impregnated intravaginal devices, and the preovulatory rise in estradiol concentrations occurred 24 h later than in the control group. However, the follicles maintained ovulatory capability. We infer that the delay in estradiol rise observed in the letrozole-treated group is responsible for the delay in ovulation in this group.

In the present study, a letrozole-impregnated intravaginal device was used to provide extended estradiol suppression during the ovulatory wave. The intravaginal route of administration provides the advantage of reducing animal handling and distress caused by daily injections [29]. The duration of estradiol inhibition was influenced by the pharmacokinetic characteristics of the intravaginal device, and accounts for the occurrence and timing of the estradiol rise observed in the letrozole-treated group. The half-life of letrozole observed following administration via an intravaginal device (33 h) corresponded to that reported previously after single intravenous administration in beef heifers (unpublished data). Hence, the profile of letrozole concentration over time obtained in the present study was affected primarily by the absorption characteristics of the formulation used in the intravaginal devices. Based on the plasma letrozole concentration profile, the intravaginal devices released letrozole for only 24 h post-insertion, and elimination and plasma clearance took place thereafter. Therefore, letrozole concentrations may have dropped below a critical level relatively rapidly, allowing for the pre-ovulatory estradiol rise to occur after only a 24 h delay. Bioavailability has been defined as the amount of a drug given by any route, other than intravenously, that reaches general circulation and is available at the site of action [30]. The low bioavailability observed with the intravaginal devices (16%) may be explained in part by the melting point of the gel-vehicle used. This gel-based vehicle is commonly used for intravaginal suppositories for women, in which body temperature is lower than that of cattle (37° vs 39°C) [31,32]. Rapid liquefaction of the letrozole-containing gel resulted in loss of the preparation through the vulvar opening during micturition, defecation, or ultrasound examinations (the latter was observed by the author).

Letrozole treatment during the growing phase of the ovulatory follicle resulted in the ovulation of a larger follicle. Although larger follicles did not result in larger CL, elevated plasma progesterone profiles were observed over the first 12 days post-ovulation in the letrozole-treated group. Preovulatory letrozole treatment may have affected the number or proportion of large luteal cells (granulosa cell origin) and small luteal cell (thecal cell origin) contained within the CL [33], resulting in an increase in progesterone production per CL volume. Small and large luteal cells are present in the bovine CL in a ratio of 7.6:1 [34]. Small luteal cells respond directly to LH stimulus to secrete progesterone [35,36], while large luteal cells appear to be responsible for sustained secretion of progesterone in the absence of a stimulus [36]. Treatment with letrozole may have resulted in an increase in luteal cell numbers or an alteration in the proportion of small and large luteal cells within the CL and an increase in progesterone-producing capability per CL volume. In this regard, treatment of cows with equine chorionic gonadotropin resulted in increased density and number of large luteal cells which increased the capacity of the CL to produce progesterone [37]. Although we were unable to document the effect of letrozole treatment on gonadotropin secretion in the present study, previous studies have shown an increase in gonadotropin secretion after single or 3-day letrozole regimen [15,38,39].

In summary, letrozole treatment during the ovulatory follicle wave resulted in more rapidly growing dominant follicles and a larger ovulatory follicles, delayed ovulation (by 24 h) of a single follicle and formation of a CL that secreted higher levels of progesterone. The effects of treatment on gonadotropin concentrations are inconclusive, possibly due to inadequate sampling frequency. However, results confirmed that letrozole treatment effectively reduces estradiol production in cattle. Finally, the formulation used for the development of an intravaginal device containing letrozole impacts on the effect of treatment on ovarian function. Based on these observations, we hypothesize that a letrozole-releasing device capable of a more sustained drug release may delay ovulation even further, while allowing more than one follicle to develop to a pre-ovulatory size when treatment is initiated prior to dominant follicle selection. The effect of estradiol suppression during the pre-ovulatory stage of follicular development on oocyte competence and fertility needs to be determined.

## Conclusions

A sustained-release intravaginal device has potential in the development of an aromatase inhibitor-based protocol for control of ovulation for herd synchronization. The enhanced effects of letrozole treatment on CL function has the potential of enhancing fertility by increasing circulating progesterone concentrations during the first 7 days post-ovulation in cattle.

## Competing interests

The authors declare that they have no competing interests.

## Authors’ contributions

JY conceived of the study, participated in its design, carried out the field experiment, radioimmunoassays, LCMS studies, statistical analysis and drafted the manuscript. RJM participated in the design of the study, and helped to draft the manuscript. RAP participated in the design of the experiment and helped draft the manuscript. JS participated in the design of the study, helped with statistical analyses. GPA conceived of the study, and participated in its design and coordination and helped to draft the manuscript. All authors read and approved the final manuscript.
